# Differentially Expressed Genes in the Pre-Eclamptic Placenta: A Systematic Review and Meta-Analysis

**DOI:** 10.1371/journal.pone.0068991

**Published:** 2013-07-12

**Authors:** C. Emily Kleinrouweler, Miranda van Uitert, Perry D. Moerland, Carrie Ris-Stalpers, Joris A. M. van der Post, Gijs B. Afink

**Affiliations:** 1 Women’s and Children’s Clinic, Department of Obstetrics, Academic Medical Center, Amsterdam, The Netherlands; 2 Reproductive Biology Laboratory, Academic Medical Center, Amsterdam, The Netherlands; 3 Bioinformatics Laboratory, Department of Clinical Epidemiology, Biostatistics and Bioinformatics, Academic Medical Center, Amsterdam, The Netherlands; VU University Medical Center, The Netherlands

## Abstract

**Objective:**

To systematically review the literature on human gene expression data of placental tissue in pre-eclampsia and to characterize a meta-signature of differentially expressed genes in order to identify novel putative diagnostic markers.

**Data Sources:**

Medline through 11 February 2011 using MeSH terms and keywords related to placenta, gene expression and gene expression arrays; GEO database using the term “placent*”; and reference lists of eligible primary studies, without constraints.

**Methods:**

From 1068 studies retrieved from the search, we included original publications that had performed gene expression array analyses of placental tissue in the third trimester and that reported on differentially expressed genes in pre-eclampsia versus normotensive controls. Two reviewers independently identified eligible studies, extracted descriptive and gene expression data and assessed study quality. Using a vote-counting method based on a comparative meta-profiling algorithm, we determined a meta-signature that characterizes the significant intersection of differentially expressed genes from the collection of independent gene signatures.

**Results:**

We identified 33 eligible gene expression array studies of placental tissue in the 3^rd^ trimester comprising 30 datasets on mRNA expression and 4 datasets on microRNA expression. The pre-eclamptic placental meta-signature consisted of 40 annotated gene transcripts and 17 microRNAs. At least half of the mRNA transcripts encode a protein that is secreted from the cell and could potentially serve as a biomarker.

**Conclusions:**

In addition to well-known and validated genes, we identified 14 transcripts not reported previously in relation to pre-eclampsia of which the majority is also expressed in the 1^st^ trimester placenta, and three encode a secreted protein.

## Introduction

Pre-eclampsia, a pregnancy-specific disorder characterized by the development of de novo hypertension and proteinuria during the second half of gestation, is a major obstetric problem that contributes substantially to maternal and perinatal morbidity and mortality worldwide [1].

The exact mechanisms involved in the development of pre-eclampsia are not well defined, but it is generally accepted that the pathogenesis of pre-eclampsia initiates at the time of trophoblast invasion and remodeling of the spiral arteries during the first and early second trimester of pregnancy. Impaired invasion of myometrial spiral arteries relates to abnormal placentation, which in turn results in the release of various components from the intervillous space into the maternal circulation. Consequently, exaggerated maternal endothelial activation and a generalized hyper-inflammatory state precede the onset of clinical symptoms of pre-eclampsia [1]–[3].

Gene expression array analysis is an important and powerful tool to identify genes differentially expressed between tissues from normotensive and pre-eclamptic pregnancies. Some key targets involved in the pathogenesis of pre-eclampsia, such as Fms-like tyrosine kinase 1 (FLT1) and Endoglin (ENG) were discovered using this approach [4], [5]. Although many studies with the aim to identify placenta-specific gene expression patterns in pre-eclampsia have been published, no clear pre-eclampsia-specific gene expression consensus signature (a list of differentially expressed genes) has been defined. This is probably due to relatively small study sample sizes, and variability in platforms, patient characteristics and statistical methods used [6].

The aim of this study was to systematically review the literature on array-based gene-expression studies of placental tissues in pre-eclampsia in order to determine a common expression signature and identify novel putative diagnostic leads.

## Methods

This systematic review was performed according to the Preferred Reporting Items for Systematic Reviews and Meta-Analyses (PRISMA) and Meta-analysis Of Observational Studies in Epidemiology (MOOSE) guidelines. [7], [8] A protocol was not specified before undertaking this systematic review.

We performed an electronic search on 11 February, 2011 in Medline (from inception) through PubMed and GEO (Gene Expression Omnibus) without language or publication date restrictions to identify all articles reporting on differentially expressed genes in human placental samples relating to pre-eclampsia.

The electronic search strategy for Medline was based on MeSH terms and keywords related to placenta, gene expression and gene expression arrays. Document S1 lists the full search strategy. Reference lists of eligible primary studies were checked to identify cited articles not captured by the electronic search. The search strategy for GEO consisted of the term “placent*”. The identified records were scanned for references not identified by the search in Medline.

### Study Selection

Eligible were those original publications that had performed gene expression array analyses of human placental tissue in the third trimester and that reported on differentially expressed genes in pregnancies that had pre-eclampsia compared to normotensive controls. Only studies presenting their results as a gene signature (defined as a list of differentially expressed genes, selected according to criteria determined by the authors of the paper) were included.

#### Studies were selected in a staged process

First, two reviewers (MM and GA) independently scrutinized titles and abstracts of all retrieved references to select potentially eligible articles. Full text papers of references selected by at least one reviewer were obtained. Second, two or three reviewers (EK, MM and GA) independently examined these full text papers to see whether they met the inclusion criteria. In case of multiple studies of the same set of women we included only the most recent or most complete one. Disagreements about inclusion were resolved by consensus.

For each study, data on clinical characteristics of included women (age, obstetric history, parity), diagnostic criteria for pre-eclampsia, characteristics of the gene expression array experiment (tissue, platform, details on sampling and preparation including extraction of RNA), details of the statistical analysis, the gene signature and other experimental results were extracted independently by two experienced reviewers (EK and MM) using standardized data extraction forms.

### Quality Assessment

As there are no formal quality assessment approaches for this type of study, we assessed study quality by discussing reported details of sampling, testing and analysis processes among four of the authors (EK, MM, CR and GA). Attention was paid to the results presented and whether the reported list of differentially expressed genes had been subject to additional selection, such that not the whole list was reported.

Acceptable diagnostic criteria for pre-eclampsia were persistent high systolic (≥140 mm Hg) or diastolic (≥90 mm Hg) blood pressure combined with proteinuria (≥0.3 g/24 hours or a dipstick result of ≥1+, equivalent to 30 mg/dL in a single urine sample or spot urine protein/creatinine ratio ≥30 mg protein/mmol creatinine) of new onset after 20 weeks of gestation, according to the International Society for the Study of Hypertension in Pregnancy criteria [9].

### Data Synthesis

From each included paper we extracted the published gene signature and selected the official gene symbol as gene identifier. If the gene symbol was not given, we mapped the other gene information (accession number or GenBank number, gene name, probe ID and UniGene ID) to the official gene symbol using the NCBI Gene database. We checked for each symbol whether this was an official symbol or an alias or both. If the symbol was not an official symbol but only an alias, we used the other gene information to obtain the official symbol.

On two occasions, multiple papers were published on the same set of placental samples. In four papers by Pang and Xing [10]–[13] various gene expression platforms targeting different, but relatively small subsets of the whole genome were used on the same samples to report on differentially expressed genes from different biological processes (for example: metabolism-related genes, apoptosis-related genes, trophoblast-invasion-associated genes). Järvenpää *et al.* [14], [15] presented partial data of the same gene expression analysis in two separate papers. In both cases we created one signature per set of samples consisting of the combined results from the different papers.

Using all gene signatures presented in the included papers, we determined a significant meta-signature using a vote counting approach based on a modified version of the so-called comparative meta-profiling method [16]. This method identifies the significant intersection of multiple gene expression signatures from a collection of datasets and consists of four steps: *Step 1:* For each gene mentioned, we counted how often it appeared in a signature and ordered the genes by number of appearance. This way we obtained (N_1_, …, N_S_), with N_x_: the number of genes that appear x times in a signature, and S: the total number of signatures. *Step 2:* We created a random signature as follows. For each signature, the number of genes remained the same as in the original signature, but the gene symbols were replaced by symbols randomly drawn from a (selected) list of 7000 official gene symbols, representing the intersection of all genes targeted by the whole genome platforms included in the meta-analysis of which the NCBI GeneID is publicly available. For the microRNA data we took a set of 854 publicly available annotated microRNAs. For these random signatures we repeated Step 1, and obtained (E_1_, …, E_S_), with E_x_: the number of genes that appear x times in a random signature. *Step 3:* Step 2 was repeated 1000 times. *Step 4:* For each *i = *1, …, S, we computed the empirical probability distribution function for E_i_+…+E_S_. Then we determined the significance of the observed number of genes present in at least i signatures, N_i_+…+N_S_. The associated p-value is the probability of E_i_+…+E_S_ ≥ N_i_+…+N_S_, reflecting the probability that the number of genes present in at least i random signatures is at least as large as the observed number. If there was an *i* such that the p-value was <0.05, then a significant meta-signature was found. If there were multiple *i* for which the p-value was <0.05, we took the smallest *i*. The genes forming the meta-signature were listed.

We performed the analyses twice: once on those studies that presented the complete list of all genes identified as differentially expressed, and once on all studies, thereby also including studies that reported an incomplete (partial) gene signature. All analyses were performed using R version 2.14.0 (The R Foundation for Statistical Computing, Vienna, Austria).

Functional interaction networks of proteins encoded by the genes in the meta-signature were visualized using STRING 9.05 [17].

## Results

**Figure 1:**
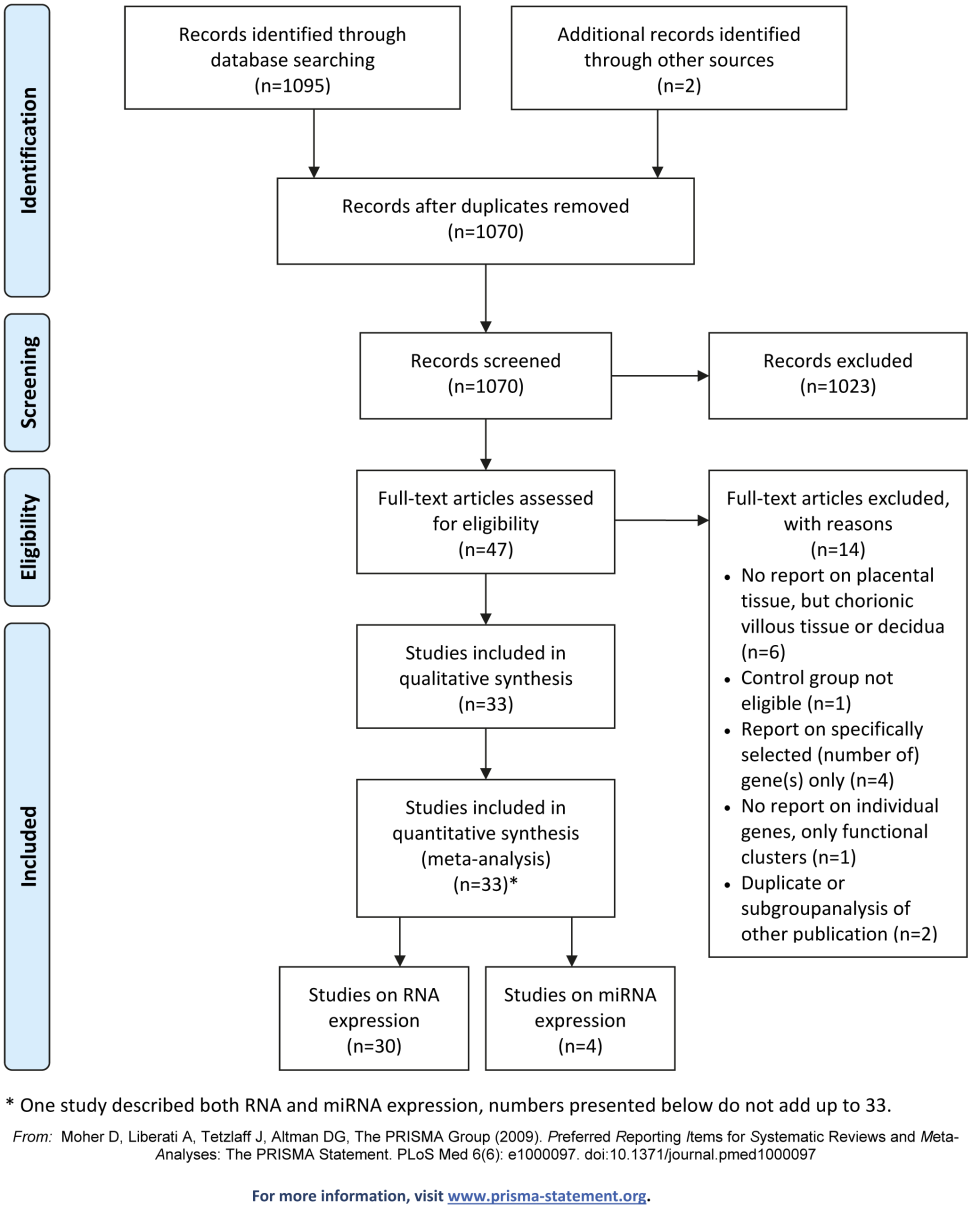
PRISMA flowchart summarizing the identification and selection of studies for inclusion in this systematic review.

The results of the search and the selection process are shown in Figure 1: The search in Medline provided a total of 1030 citations. In addition, the search in GEO yielded 67 eligible datasets, 65 with a publication, of which 38 publications had not been identified by the Medline search. Of the total of 1068 unique citations, 1023 studies were discarded after reviewing titles and abstracts. The full text of the remaining 45 papers was examined in more detail. Of these, 14 studies did not meet the inclusion criteria as described (see Document S2). Two new studies where identified from reference lists of eligible studies and by keeping up with the literature. Finally, 33 studies met the inclusion criteria and were included in this systematic review. All studies reported on third trimester placental samples of which 30 studies determined the mRNA expression profile [10]–[15], [18]–[41] and 4 studies determined the microRNA expression profile [30], [42]–[44]. All studies were published between 2002 and 2011. Study sample size ranged from 3 to 62 included women. We provide an overview of all studies in Table S1.

### Quality

Tables S1 and S2 summarize the details of the study characteristics, as described in the original papers, that we obtained for quality assessment. Overall, the impression of study quality was fair and we did not exclude any studies for poor quality. Most studies had adequately reported an acceptable definition of pre-eclampsia used in the study, the type and location of tissue sampling, the storage and RNA isolation conditions, and the array platform used. Most samples were stored in liquid nitrogen or RNAlater (Qiagen, Valencia, USA or Ambion, Austin, USA), and most studies used either RNeasy (Qiagen, Valencia, USA) or Trizol (Invitrogen, Carlsbad, USA) for RNA isolation. Gene expression array platforms were very diverse, ranging from whole genome arrays to nylon membrane hybridizations, most of them commercially available. Most studies had a case-control design with an appropriate control group and adequately reported characteristics of the case and control women (age, parity, pregnancy details and gestational age at delivery). The major obstacles in the quality assessment were the biased listing of incomplete gene signatures and the diversity in statistical procedures used (normalization and analysis methods).

### Meta-profiling

Fourteen third-trimester placental tissue RNA expression studies that reported a complete gene signature were included in the meta-analysis. These studies represented 159 samples of pre-eclamptic placenta and 188 control samples. The case where a gene was present in 3 or more of 14 complete gene signatures turned out to be significant (p<0.001). According to this criterion, 17 of the 840 genes present in at least one signature were incorporated in the meta-signature. Most genes were up-regulated in pre-eclampsia compared to normotensive placentas (Table 1, Table S3). The meta-analysis with complete and incomplete signatures combined covered a total of 254 pre-eclampsia samples and 278 control samples from 30 studies. As the four studies from Pang and Xing were considered to form one signature, as were the two studies by and Järvenpää *et al.*, in total 26 gene signatures were included in the analysis. A gene had to be present in 3 or more of these 26 signatures in order to be part of the significant meta-signature (p<0.001). This way, 40 of 1343 genes were incorporated in the meta-signature, in which a large majority of genes was up-regulated in pre-eclampsia (Table 1, Table S3).

**Table 1:** Meta signature of third-trimester placental tissue mRNA expression.

Gene symbol	Encodes extracellularprotein	Number of times present incomplete signature	Number of times present in any signature	Expression inpre-eclampsia
LEP	+	5	12	Up
FLT1	+	4	11	Up
INHBA	+	5	9	Up
ENG	+	5	8	Up
EBI3	+	3	6	Up*
INHA	+	4	6	Up
SIGLEC6	+	3	6	Up
BCL6		3	5	Up
CGB	+	*2*	5	Up
CRH	+	*2*	5	Up*
HTRA1	+	3	5	Up
PAPPA2	+	3	5	Up*
CYP11A1		3	4	Up*
FSTL3	+	*1*	4	Up
KRT19		*2*	4	Up
SLCO2A1		*2*	4	Up
SOD1	+	3	4	–
AQP1		*2*	3	Up*
BHLHE40		*1*	3	Up
CGA	+	*1*	3	Up
EZR		*1*	3	Up
F5	+	*2*	3	Down
HEXB		*1*	3	Up
HSD17B1		*2*	3	Down
HTRA4	+	*1*	3	Up
IGFBP1	+	*1*	3	Up*
LHB	+	*2*	3	Up
PGF	+	*2*	3	Up*
PHYHIP		3	3	Up
PLEC		3	3	Up
PVRL4	+	*1*	3	Up
RDH13		3	3	Up
SASH1		3	3	Up
SEMA4C		*2*	3	Up
SPAG4		3	3	Up
SPP1	+	*2*	3	Up
TNFSF10	+	*0*	3	Up
TREM1	+	*1*	3	Up
VEGFA	+	*1*	3	Up*
VIM		*1*	3	–

Genes present in 3 or more complete signatures, or 3 or more signatures in total, are part of the meta-signature. For comparison, the number of times a gene is present in complete signatures below the significance threshold is shown in italics. Enquobahrie 2008, Kang 2011, Pang (combined) and Vaiman 2005 did not report the direction of differential expression (upregulation or downregulation).

* Results were inconsistent among studies, the majority of studies reported the result presented here.

–No consensus among studies: equal numbers of studies reported up- and downregulated expression in PE.

The 4 studies on third-trimester placental tissue microRNA expression represented 39 pre-eclampsia samples and 38 control samples. A gene had to be present in 2 or more signatures in order to be part of the significant meta-signature (p = 0.001) resulting in a meta-signature consisting of 17 out of 127 miRNAs (Table 2).

**Table 2:** Meta signature of 4 studies on third-trimester placental tissue microRNA expression.

miRNA name	Present in number of publications	Expression inpre-eclampsia	References
hsa-miR-126	3	Down*	Hu 2009, Mayor-Lynn 2011, Zhu 2009
hsa-miR-210	3	Up	Enquobahrie 2011, Mayor-Lynn 2011, Zhu 2009
hsa-miR-584	3	Down*	Enquobahrie 2011, Mayor-Lynn 2011, Zhu 2009
hsa-miR-1	2	Down	Enquobahrie 2011, Zhu 2009
hsa-miR-139-5p	2	Down	Enquobahrie 2011, Mayor-Lynn 2011
hsa-miR-150	2	Down	Mayor-Lynn 2011, Zhu 2009
hsa-miR-181a	2	Up	Hu 2009, Zhu 2009
hsa-miR-195	2	–	Hu 2009, Zhu 2009
hsa-miR-26b	2	–	Hu 2009, Mayor-Lynn 2011
hsa-miR-27a	2	–	Hu 2009, Mayor-Lynn 2011
hsa-miR-377	2	–	Mayor-Lynn 2011, Zhu 2009
hsa-miR-423-5p	2	–	Hu 2009, Mayor-Lynn 2011
hsa-miR-519b-3p	2	–	Hu 2009, Mayor-Lynn 2011
hsa-miR-519e	2	–	Mayor-Lynn 2011, Zhu 2009
hsa-miR-542-3p	2	Down	Mayor-Lynn 2011, Zhu 2009
hsa-miR-625	2	Down	Mayor-Lynn 2011, Zhu 2009
hsa-miR-638	2	Up	Mayor-Lynn 2011, Zhu 2009

MicroRNAs incorporated in the meta-signature are ordered by (1) the number of publications that reported the miRNA as differentially expressed and (2) alphabetically.

* Results were inconsistent among studies, the majority of studies reported the result presented here.

–No consensus among studies: 1 study reported upregulated expression and 1 study reported downregulated expression in pre-eclampsia.

### Interaction Network Analysis

**Figure 2:**
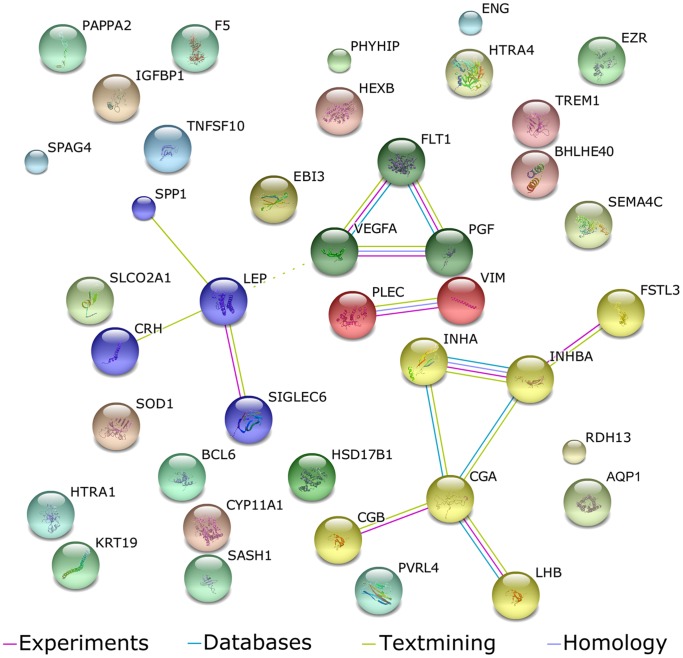
Functional networks within the meta-signature. To build a functional network map the 40 meta-signature genes were analyzed using STRING software. Interactions are based on information from experimental and curated protein databases, and text mining with a high confidence setting of 0.8; the resulting networks were clustered using Markov clustering.

Using high confidence scores based on experimental data and associations in curated protein databases and text mining, 15 of the 40 signature genes could be mapped into an interaction network (Figure 2). Subsequent clustering identified at least four different functional clusters. In addition, 60% of the gene products are secreted by cells, according to the DAVID functional annotation tool [45], and therefore potentially detectable in the maternal circulation (Table 1, Table S3).

## Discussion

The placenta is the key organ involved in pre-eclampsia. Identification of genes differentially expressed between normotensive and pre-eclamptic placentas is essential to understand the molecular mechanisms involved in the etiology of the disease and to develop novel biomarkers or identify therapeutic targets. This systematic review provides an overview of placental tissue gene expression array studies in pre-eclampsia. It is the first study that extracts a meta-signature from these individual studies consisting of up-regulated and down-regulated genes in pre-eclampsia as identified in multiple publications.

When all 30 studies generating signatures on placental tissue mRNA expression are included, the pre-eclamptic placental meta-signature consists of 40 genes. As authors reporting an incomplete list of genes may be driven by bias towards the genes they list (for instance only specific genes of the authors’ particular interest), we also generated a meta-signature from the 14 complete gene-lists by which we obtained a meta-signature of 17 genes. As the ratio of the number of times a gene is present in a signature compared to the total number of datasets is similar for both meta-signatures we conclude that the inclusion bias does not affect the overall meta-signature very strongly.

Both meta-signatures have identical well-known and validated genes on top of their list. The relation of the angiogenesis-related cluster FLT1, VEGFA, and PlGF to pre-eclampsia has been extensively documented. Members of this cluster are used as biomarkers and have been shown to play a functional role in pre-eclampsia [46]–[48]. Also the clusters around INH(B)A, CGA/CGB, and LEP have previously been reported to be affected in the pre-eclamptic placenta and some cluster members have been evaluated in the circulation of affected women [49]–[56].

In both the meta-signature derived from complete and incomplete signatures, the most frequently occurring genes are only mentioned in approximately one third of the individual signatures. Variation in technical aspects, as reported in Tables S1 and S2, may contribute to inconsistencies. As studies were published between 2002 and 2011, the data also reflect the development of more sophisticated array techniques in this decade, including an increased number of genes interrogated and improved gene annotations. A major shortcoming is the variability between studies in statistical methods used for detecting differentially expressed genes. We repeatedly encountered inadequate reporting of statistical procedures and their results, and some studies used inappropriate or not stringent enough statistical criteria. However, as our comparative meta-profiling method extracts only results that are relatively consistent across studies, the contribution of individual biased results is limited.

There was good overall agreement on the direction of differential gene expression among studies. An exception seems to be the data of Mayor-Lynn *et al*. [30] that show a deviant direction for CRH, EBI3, PAPPA2, and CYP11A1 compared to the other studies. For CRH, the authors report a more than 10-fold down-regulation of CRH mRNA by microarray analysis in pre-eclamptic placentas while subsequent RT-QPCR in the same tissue shows a 5-fold up-regulation of CRH mRNA. As the raw data are not publically available, the reason for this discrepancy is untraceable.

Apart from transcripts previously related to pre-eclampsia, the meta-signature includes 14 genes for which no information with regard to pre-eclampsia has been reported. They encode structural proteins (KRT19, PLEC, SPAG4, VIM), plasma membrane proteins with a channel/transporter or receptor function (AQP1, PVRL4, SEMA4C, SLCO2A1), proteins involved in immunomodulation (EBI3, TNFSF10), a mitochondrial protein (RDH13), a nuclear protein (BHLHE40) and cytosolic proteins (PHYHIP, SASH1). With the exception of VIM, the expression of all these genes is up-regulated in pre-eclamptic placenta.

Interestingly, 3 of these 14 genes encode a secreted protein that might be detectable in the maternal circulation. However, as the placenta deports large amounts of cellular material into the maternal circulation, non-secreted proteins should not be excluded as candidate biomarkers [57], [58]. Therefore, placental expression in early pregnancy may be more relevant, but available information on this condition is very limited. There are two studies reporting mRNA expression data of chorionic villous samples from women that developed pre-eclampsia later during their pregnancy. Although one study [59] reports two differentially expressed genes (FSTL3 and IGFBP1) that are also included in our meta-signature, these genes are not reported in the other study [60]. Analysis of the data from the first study [59] combined with data from 1^st^ trimester placental mRNA expression [61] shows that 10 of the 14 genes (that are novel in relation to pre-eclampsia) included in our meta-signature are expressed in the early placenta (Table S3).

Due to the limited number of available studies, meta-analysis of the microRNA expression studies resulted in a less robust meta-signature. In particular the direction (up/down) of the microRNA expression showed a high level of ambiguity between studies. As microRNA expression is considered a regulatory layer for silencing gene expression predominantly by degradation of their target mRNAs [62], our finding of overall down-regulation of microRNA expression in pre-eclamptic placenta supports our results that most gene transcripts in the meta-signature are up-regulated.

Meta-analysis of raw whole genome expression data would partly overcome the methodological variation encountered in this paper and should be undertaken to validate and extend the novel leads with respect to diagnosis and putative therapeutic targets identified here.

In addition, the combined analysis of microRNA and mRNA meta-signatures is a further necessary step to comprehend the overall deviating transcription profile of the pre-eclamptic placenta.

## Supporting Information

Table S1 **Clinical data: characteristics of all studies and women included in this systematic review and meta-analysis, as they were reported in the original papers.** Data on maternal age, parity and gestational age at delivery are reported as mean (standard deviation) or median (range), unless indicated otherwise. FGR: fetal growth restriction; IUGR: intra uterine growth restriction; PE: pre-eclampsia.
(DOCX)

Table S2 **Experimental data: details of the sampling, testing and analysis processes of all studies, as they were reported in the original papers.** ANOVA: analysis of variance; PE: pre-eclampsia. ^a^: A complete list is defined as the complete list of genes identified as differentially expressed according to the studies’ selection criteria.
(DOCX)

Table S3 **Meta signature of third-trimester placental tissue mRNA expression.** Genes present in 3 or more complete signatures, or 3 or more signatures in total, are part of the meta-signature. Genes incorporated in the meta-signature are ordered by (1) the total number of publications that reported the gene as differentially expressed and (2) alphabetically. References printed in bold indicate publications that reported a complete signature. Enquobahrie 2008, Kang 2011, Pang (combined) and Vaiman 2005 did not report the direction of differential expression (upregulation or downregulation). For comparison, the number of times a gene is present in complete signatures below the significance threshold is shown in grey. *: Results were inconsistent among studies, the majority of studies reported the result presented here. –: No consensus among studies: equal numbers of studies reported upregulated expression and downregulated expression in pre-eclampsia. 1^st^ trimester expression was scored using microarray data from control chorionic villous sampling (NCBI Gene Expression Omnibus GSE12767) and 1^st^ trimester placenta samples (NCBI Gene Expression Omnibus GSE9984). Microarrays were analyzed for expression using The Gene Expression Barcode 2.0 (http://barcode.luhs.org/). ‘+’ indicates that in >90% of the samples expression of the gene was detected, ‘–’ indicates that in <10% of the samples expression of the gene was detected. The remaining samples showed a variable degree of expression (‘+/−’).
(DOCX)

Document S1 **Electronic search strategy PubMed.**
(DOC)

Document S2 **Studies that were excluded after screening of full text and new studies that were included, as described in** **Figure 1** **.**
(DOC)

Document S3 **PRISMA 2009 Checklist.**
(DOC)
