# Supplementary material for: Differentially Expressed Genes in the Pre-Eclamptic Placenta: A Systematic Review and Meta-Analysis
Source: PLoS One. 2013 Jul 12;8(7):e68991. doi: 10.1371/journal.pone.0068991 (PMC3709893; doi:10.1371/journal.pone.0068991)
Supplement: Table S1 — Clinical data: characteristics of all studies and women included in this systematic review and meta-analysis, as they were reported in the original papers. Data on maternal age, parity and gestational age at delivery are reported as mean (standard deviation) or median (range), unless indicated otherwise. FGR: fetal growth restriction; IUGR: intra uterine growth restriction; PE: pre-eclampsia. (DOCX) [file pone.0068991.s001.docx]

**Table S1. Clinical data: characteristics of all studies and women included in this systematic review and meta-analysis, as they were reported in the original papers**

**Part 1: Studies on third-trimester placental tissue RNA expression**

| **First author,**  **year** | **Country of data collection** | **Population PE** | **Population controls** | **Maternal age** | **Parity** | **Gestational age at delivery** | **Definition PE** | **#PE** | **#controls** |
| --- | --- | --- | --- | --- | --- | --- | --- | --- | --- |
| Ahr 2006 | Germany | Women with severe PE and IUGR | Women with uncomplicated pregnancy and delivery | PE 32 (32-36), controls 38 (26-39) | PE all nulliparous, controls 0.5 (0-1) | PE 32 (32-36), controls 39 (38-39) | Hypertension (>140/90 mm Hg) and proteinuria (>0.3 g/24h) | 3 | 4 |
| Centlow 2011 | Sweden | Women with PE | Control women | PE 29 (22-34), controls 31 (24-37) | Not reported | PE 37 (32-41), controls 40 (36-42) | Blood pressure above 140/90mmHg and proteinuria above 0.3 g/l | 10 | 15 |
| Enquobahrie 2008 | USA | Participants of Omega study (women who initiated prenatal care before 16 weeks of gestation) and the Placenta MicroArray study (women who delivered at Swedish Medical Center) without a history of chronic hypertension and/or pregestational diabetes mellitus with current singleton pregnancies | Women with normotensive pregnancies uncomplicated by proteinuria or gestational diabetes | PE 32, controls 30 ^#^ | PE 72.2% nulliparous, controls 55.6% | PE 35.8, controls 38.9 ^#^ | Sustained (≥ 2 measures 6 hours apart) blood pressure elevation (>140/90mmHg) after 20 weeks of gestation and sustained (≥ 2 measures 4 hours apart) proteinuria (>30 mg/dL or dipstick >1+) | 18 | 18 |
| Gack 2005 | Germany | Women with pregnancies complicated by PE with no other underlying clinical problems, such as chorioamnionitis, other infections, or renal diseases. Included were one case with twin pregnancy and two cases with also IUGR | Women with pregnancies with no hypertension matched according to their gestational ages | PE 31 (28-40), controls 33 (23-38) | PE 0 (0-2), controls 1 (0-3) | PE 36 (25-41), controls 36 (27-41) | Blood pressures of ≥140/90 mmHg measured twice within an interval of ≥ 6 h and proteinuria (3+ or ≥300 mg in 24-h urine collection) in women who were normotensive before pregnancy | 4 | 4 |
| Han 2006 | Korea | Women with PE without chronic hypertension, (pre-)gestational diabetes, chronic renal disease or cardiac disease | Women without evidence of chronic hypertension, PE, or gestational hypertension | PE 32 (3.3), controls 30 (2.8) | Not reported | PE 36 (1.6), controls 38 (2.0) | High blood pressure (systolic >160 mmHg or diastolic >90 mmHg) associated with proteinuria (≥300 mg in 24-hours urine) in women at ≥20 weeks of gestation | 8 | 11 |
| Hansson 2006 | Sweden | Caucasian women with PE without essential hypertension or renal or other systemic diseases | Healthy women, one black and the others Caucasian | PE 32 (23-40), controls 32 (28-38) | Not reported | PE 37 (26-40), controls 39 (36-40) | Blood pressure 140/90 mmHg or a rise in blood pressure of 20 mmHg as compared with the first trimester of pregnancy, and proteinuria 0.3 g/L | 9 | 7 |
| Heikkilä 2005 | Finland | Women having Cesarean sections due to severe PE | A healthy woman with dizygotic twin-pregnancy having Cesarean section due to cardiac problems of one fetus. Control samples of placental tissue are from the healthy twin | PE 24-32, control 36 | PE all nulliparous, control para 1 | PE 25-27, control 29 | According to the report of the National High Blood Pressure Education Program Working Group on High Blood Pressure in Pregnancy | 2 | 1 (placenta from healthy twin) |

**Part 1 (continued)**

| **First author,**  **year** | **Country of data collection** | **Population PE** | **Population controls** | **Maternal age** | **Parity** | **Gestational age at delivery** | **Definition PE** | **#PE** | **#controls** |
| --- | --- | --- | --- | --- | --- | --- | --- | --- | --- |
| Herse 2007 | Norway | Nonsmokers, white, and previously healthy with uncomplicated pregnancies. None had chronic hypertension, renal disease, or diabetes. All were delivered by cesarean section. None were in active labor nor had any infection at the time of cesarean delivery. One baby was small for gestational age (<p10) | Normotensive women with uneventful pregnancy undergoing cesarean delivery because of breech presentation or other medical indications | PE 29 (19-38), controls 28 (21-37) | All nulliparous | PE 33 (26-36), controls 39 (34-39) | Rise in blood pressure after 20 weeks of gestation to >140/90 mm Hg on ≥2 occasions 6 hours apart in a previously normotensive woman, combined with proteinuria defined as a protein dipstick reading of ≥1+ or a 24-hour urinary excretion of ≥0.3 g protein in ≥2 urine samples, in the absence of urinary tract infection | 10 | 10 |
| Hoegh 2010 | Denmark | Non-smoking women with pregnancies affected by PE. Most cases had vaginal deliveries | Non-smoking women with uncomplicated pregnancies, matched for maternal age, parity, gestational week at delivery, smoking, gender of the child, and when possible, method of delivery. Most controls had elective Caesarean sections | PE 29 (24-35), controls 32 (26-35) | All multiparous | PE 38 (33-40), controls 38 (35-39) | Blood pressure ≥140/90mmHg or a rise in diastolic blood pressure of ≥30mmHg, proteinuria of ≥300 mg/24 hours or ≥300 mg/L or ≥1+ by urinary dipstick analysis | 9 | 9 |
| Järvenpää 2007 | Finland | Pre-eclamptic women with IUGR without chronic hypertension or gestational diabetes who delivered by Cesarean section before labor because of imminent asphyxia | Healthy women with uncomplicated pregnancies who delivered by Cesarean section because of an earlier Cesarean or breech presentation | PE both 21, controls 27 (27-29) | Not reported | PE 35-36, controls 39 (38-39) | Blood pressure >140/90 mmHg with proteinuria >0.5 g/day. PE with IUGR is classified as severe PE | 2 | 3 |
| Järvenpää 2009 | Finland | Women with severe PE, one with intrauterine growth retardation, who had cesarean deliveries for imminent asphyxia | Healthy women with uncomplicated pregnancies in late pregnancy undergoing caesarean section because of an earlier cesarean, breech presentation or severe fear of childbirth | PE 26.9, controls 29.9 ^#^† | PE 0.23, controls 0.75 ^#^† | PE 36.0, controls 38.7 ^#^† | Blood pressure over 140/90 with proteinuria over 0.5 g per day | 2 | 3 |
| Kang 2011 | Korea | Women with PE who delivered via elective Cesarean sections without labor without a history of cardiovascular, renal, or other hypertension-associated disease. Multiple pregnancies, chromosomal disorders, congenital malformation, or suspected perinatal infections were excluded from both study groups | Women with uncomplicated pregnancies | PE 3 (3.9), controls 33 (4.2) | Not reported | PE 36 (2.4), controls 39 (0.9) | Gestational hypertension (systolic pressure >140mmHg or diastolic blood pressure >90mmHg on ≥ 2 occasions after gestational week 20) with proteinuria (>0.3 g/day) | 17 | 16 |

**Part 1 (continued)**

| **First author,**  **year** | **Country of data collection** | **Population PE** | **Population controls** | **Maternal age** | **Parity** | **Gestational age at delivery** | **Definition PE** | **#PE** | **#controls** |
| --- | --- | --- | --- | --- | --- | --- | --- | --- | --- |
| Lee 2010 | Korea | Women with severe PE who received cesarean section without active labor | Normal pregnant women with no existing medical diseases or antenatal complications who received cesarean section without active labor. Fetuses with congenital malformations, chromosomal disorders, or intrauterine infections were excluded | PE 31 (3.9), controls 33 (4.6) | PE 0.62 (0.77), controls 0.69 (0.48) | PE 35.9 (0.9), controls 38.5 (0.6) | Severe PE: blood pressure of 160/110 mmHg taken twice, 6 h apart, with proteinuria of ≥3+ or 2 g in a 24-h collection | 13 | 13 |
| Liu 2008 | China | Pregnant women with abnormal high resistance umbilical artery blood flow (systolic/diastolic ratio > 3) at 1 to 4 days prior to delivery by elective cesarean section. 18 were diagnosed with PE, 4 with IUGR, and 6 with both PE and IUGR | Uncomplicated pregnancies with no identifiable medical or obstetric diseases who were delivered by elective cesarean section at term (for reasons not associated with fetal compromise) | Not reported | Not reported | PE 35 (2.8), controls 38 (2.0) | Hypertension (systolic ≥ 140 mmHg or diastolic ≥ 90 mmHg), with onset after the 20^th^ week of pregnancy, accompanied by proteinuria (> 300 mg/24 h) | 28 | 34 |
| Mayor-Lynn 2011 | USA | Women with PE without diabetes mellitus, preterm premature ruptured membranes without labor, multiple gestations, and fetal demise in utero or fetal anomalies | Women who delivered normal infants at term without labor via elective cesarean section | PE 23 (20-26), controls 30 (21-38)* | PE 2/7 nulliparous, controls 1/7* | PE 35 (31-39), controls 38 (37-39)* | Hypertension (systolic ≥140 mm Hg, or diastolic ≥90 mmHg, on at least 2 occasions, at least 4 hours apart, and proteinuria of ≥300 mg in a 24-hour urine collection or urine dipstick measurement of ≥ 2+ | 6 | 6 |
| Nishizawa 2011 | Japan | Women with severe PE who had not undergone labor and underwent Caesarean sections | Normotensive women with or without FGR, matched for maternal and gestational ages and for body mass index during pre-pregnancy, in whom Caesarean sections were performed due to previous Caesarean sections | PE 31 (4.7), controls 31 (6.5) | Not reported | PE 34.4 (1.8), controls 38.1 (0.8) | Blood pressure higher than 140/90 mmHg, with proteinuria of more than 0.3g in a 24 hour collection. PE was considered severe if blood pressure was higher than 160/110 mmHg, and/or proteinuria more than 2g in a 24 hour collection | 8 | 8 |
| Nishizawa 2007 | Japan | Women with PE who underwent Caesarean sections without labor | Normotensive women were matched for maternal and gestational ages, and for body mass index during pre-pregnancy | PE 30.3 (3.7), controls 29.2 (5.9) ‡ | Not reported | PE 32.9 (4.0), controls 32.9 (5.6) ‡ | Blood pressure higher than 160/110 mmHg, with proteinuria of more than 2 g in a 24 h collection | 10 | 4 |
| Pang 2003 Br J Biomed Sci | China | Women with PE without a history of cardiovascular, renal or other hypertension-associated diseases | Women with normal pregnancies strictly matched for age (24/25 years old), weight and body shape | All 24-25 years old | Not reported | PE not reported, controls all >37 | At least three new-onset symptoms including blood pressure (≥140/ 90 mmHg), proteinuria (≥2+) and edema after 20 gestational weeks | 5 | 5 |

**Part 1 (continued)**

| **First author,**  **year** | **Country of data collection** | **Population PE** | **Population controls** | **Maternal age** | **Parity** | **Gestational age at delivery** | **Definition PE** | **#PE** | **#controls** |
| --- | --- | --- | --- | --- | --- | --- | --- | --- | --- |
| Pang 2003  J Perinat Med | China | Women with PE who delivered by caesarean section and did not experience a hard 24 h labor before delivery with or without MgSO4. Women had no history of cardiovascular, renal or other hypertension-associated diseases | Women with normal, term pregnancies strictly matched according to age (24/25 years old), weight, body shape and partial method | All 24-25 years old | All primiparous | All >37 | At least three new-onset symptoms including blood pressure (≥140/ 90 mmHg), proteinuria (≥2+) and edema after 20 gestational weeks | 5 | 5 |
| Pang 2004  J Perinat Med | China | Women with PE without history of cardiovascular, renal or other hypertension-associated diseases. | Women with normal, term pregnancies strictly matched according to age (24/25 years old), weight, body shape and partial method | All 24-25 years old | All primiparous | All >37 | At least three new-onset symptoms including blood pressure (≥140/ 90 mmHg), proteinuria (≥2+) and edema after 20 gestational weeks | 5 | 5 |
| Pang 2004 Arch Gynecol Obstet | China | Women with PE with spontaneous deliveries without history of cardiovascular, renal or other hypertension-associated diseases | Women with normal, term pregnancies strictly matched according to age (24/25 years old), weight, body shape and partial method | All 24-25 years old | All primiparous | All >37 | At least three new-onset symptoms including blood pressure (≥140/ 90 mmHg), proteinuria (≥2+) and edema after 20 gestational weeks | 3 | 3 |
| Reimer 2002 | Germany | Women with PE and delivery ≤32 weeks | Normotensive women matched for maternal and gestational ages, and for pre-pregnancy body mass index | PE 27 (20-31), controls 25 (19-32) | PE all nulliparous, controls 0 (0-3) | PE 31 (29-32), controls 32 25-32 | Blood pressure of ≥140/90 mmHg taken twice, 6h apart, with proteinuria of ≥2+ or ≥300 mg in a 24h collection | 6 | 6 |
| Sitras 2009 | Norway | Caucasian women with severe PE, of which 2 women had IUGR babies, and abnormal umbilical artery Doppler. 11/16 women had caesarean sections. Women with pre-existing chronic hypertension, renal disease, lupus erythematosus, diabetes, and gestational hypertension without proteinuria were excluded | Healthy women without pre-existing conditions and with uncomplicated pregnancies, matched for parity, randomly selected out of 50 women. 8/21 women had caesarean sections | PE 30 (5.2), controls 30 (4.8) | PE 7/16 nulliparous, controls 9/21 nulliparous | PE 34 (3.6), controls 39 (1.3) | Severe PE: systolic blood pressure ≥ 160 mmHg and/or diastolic ≥110 mmHg, with proteinuria ≥2+ on dipstick, measured on at least two occasions 6 h apart while the patient was on bed rest, or hemolysis, elevated liver enzymes and low platelet (HELLP) syndrome, after the 20th week of gestation | 16 | 21 |
| Soleymanlou 2005 | Canada | Women with PE including two cases complicated by IUGR, and one complicated by hemolysis elevated liver enzymes and low platelets, 10 nonlabor and one in labor | Normotensive, age-matched women with preterm deliveries, four multiplets and five preterm labor, all in labor | PE 28 (6.0), controls were age-matched, data not reported | Not reported | PE 29 (25-34), controls not reported | Classic severe early-onset PE according to both clinical and pathological criteria based on the ACOG 2002 criteria | 11 | 9 |

**Part 1 (continued)**

| **First author,**  **year** | **Country of data collection** | **Population PE** | **Population controls** | **Maternal age** | **Parity** | **Gestational age at delivery** | **Definition PE** | **#PE** | **#controls** |
| --- | --- | --- | --- | --- | --- | --- | --- | --- | --- |
| Tsai 2011 | USA | Women aged 18 and older with PE. Women with multiple gestations, fetuses with documented congenital or chromosomal anomalies, and fetuses with prolonged premature rupture of the membranes (> 4 weeks) were excluded | Control women according to the same criteria | Not reported | PE 0 (0-2), controls 2 (0-7) | PE 33 (27-38), controls 37 (29-40) | Systolic blood pressure ≥140 mm Hg and diastolic blood pressure ≥ 90 mm Hg and proteinuria with ≥300 mg of protein in 24 h measured directly or indirectly by protein creatinine ratio | 23 | 37 |
| Tsoi 2003 | USA | Non-laboring women at term with PE who had cesarean deliveries | Non-laboring women at term with normal pregnancies who had cesarean deliveries | Not reported | Not reported | Term, not otherwise reported | Onset of hypertension during late pregnancy with systolic and diastolic blood pressure greater than 140/90 mm Hg on at least two occasions and urinary protein greater than 2+ on dipstick or greater than 0.3 g/24 hours | 4 | 4 |
| Vaiman 2005 | France | Mothers with PE with or without IUGR who had caesarean sections outside labor | Healthy mothers who had caesarean sections outside labor | PE 36 (22-45), controls 37 (32-43) | Not reported | PE 32 (27-37), controls 38 (38-40) | Not reported | 9 | 7 |
| Várkonyi 2011 | Hungary | Women with early-onset PE (≤ 35 weeks) who had caesarean sections due to severe symptoms. Patients with multiple pregnancies or fetuses having congenital or chromosomal abnormalities were excluded. Severe preeclampsia was diagnosed in 66.7% of women | Term (n= 5; >37 weeks) and preterm controls (n=5; ≤35 weeks) combined who had caesarean sections due to previous caesareans or malpresentation. Term controls had no medical or obstetrical complications and delivered a neonate with a birth-weight appropriate for gestational age. Preterm controls delivered preterm without clinical or histological signs of chorioamnionitis | PE 34 (30-35), controls 34 (30-34) | PE 66.7% primiparous, controls 40% primiparous | PE 32 (30-34), controls 36 (31-38) | Defined according to the criteria set by ACOG Bulletin 2002 | 6 | 10 |
| Winn 2009 | USA | Women whose singleton pregnancies were complicated by PE.  Pregnancies complicated by fetal anomalies, premature rupture of the membranes, infection, diabetes, or other autoimmune diseases were excluded | Women with singleton pregnancies who experienced preterm labour without signs of infection, absence of premature rupture of membranes, and no significant maternal disease, who were matched for gestational age at delivery. Approximately one third of the women delivered due to cervical insufficiency | PE 30 (9.1), controls 30 (7.1) | PE 9/12 nulliparous, controls 7/11 nulliparous | PE 32 (3.3), controls 31 (4.6) | As defined in a 2002 ACOG bulletin | 12 | 11 |

**Part 1 (continued)**

| **First author,**  **year** | **Country of data collection** | **Population PE** | **Population controls** | **Maternal age** | **Parity** | **Gestational age at delivery** | **Definition PE** | **#PE** | **#controls** |
| --- | --- | --- | --- | --- | --- | --- | --- | --- | --- |
| Zhou 2006 | China | Women with mild PE who delivered by cesarean section before labor. Women whose neonates had dysmorphic features, congenital malformations, intrauterine infections, or organic or chromosomal disorders were excluded | Pregnant women with no preexisting medical diseases or antenatal complications who delivered by cesarean section before labor | PE 25 (24-29), controls 27 (24-29) | Not reported (no significant difference between PE and controls in terms of gravidity) | PE 38 (37-39), controls 38 (38-39) | Onset of hypertension during late pregnancy with systolic and diastolic blood pressure greater than 140/90 mm Hg on at least two occasions and urinary protein greater than 2+ on dipstick or greater than 0.3 g/24 h | 5 | 5 |

^#^: data presented as mean.

†: these data are reported for a larger group of women (cases n=13 and controls n=16) that includes the women in the microarray experiments.

*: these data are reported for a larger group of women (cases n=7 and controls n=7) that includes the women in the microarray experiments.

‡: these data are reported for a larger group of women (cases n=21 and controls n=24) that includes the women in the microarray experiments.**Part 2: Studies on third-trimester placental tissue microRNA expression**

| **First author,**  **year** | **Country of data collection** | **Population PE** | **Population controls** | **Maternal age** | **Parity** | **Gestational age at delivery** | **Definition PE** | **#PE** | **#controls** |
| --- | --- | --- | --- | --- | --- | --- | --- | --- | --- |
| Enquobahrie 2011 | USA | Participants of Omega study (women who initiated prenatal care before 16 weeks of gestation) and the Placenta MicroArray study (women who delivered at Swedish Medical Center) without a history of chronic hypertension and/or pregestational diabetes mellitus and current singleton pregnancies | Controls were frequency matched for parity, maternal ethnicity, and labor status | PE 32 (7.4), controls 30 (5.6) | PE 65% nulliparous, controls 60% | PE 36.0 (0.9), controls 38.8 (0.3) | Sustained (≥ 2 measures 6 hours apart) blood pressure elevation (>140/90mmHg) after 20 weeks of gestation and sustained (≥ 2 measures 4 hours apart) proteinuria (>30 mg/dL or dipstick >1+) | 20 | 20 |
| Hu 2009 | China | Chinese women with pregnancies complicated by severe late-onset PE with delivery occurring after 34 weeks | Women with normal term pregnancies without chronic hypertension, cardiovascular disease, renal disease, hepatitis, diabetes, any evidence of intrapartum infection or other pregnancy complications, such as fetal anomalies or chromosomal abnormalities. Controls were matched for gestational age at delivery and maternal age | PE 28.1 (6.4), controls 28.7 (5.6) † | PE 10/24 primiparous, controls 15/26 † | PE 37.0 (1.0), controls 38.8 (2.0) † | Gestational hypertension (systolic >140 mm Hg or diastolic pressure >90 mm Hg on ≥2 occasions after gestational week 20) with proteinuria (>0.3 g/day). Severe PE was defined by severe gestational hypertension (systolic >160 mm Hg or diastolic >110 mm Hg on ≥2 occasions after gestational week 20); and/or severe proteinuria (≥5 g protein in a 24-h urine specimen) | 4 | 4 |
| Mayor-Lynn 2010 | USA | Women with PE without diabetes mellitus, preterm premature ruptured membranes without labor, multiple gestations, and fetal demise in utero or fetal anomalies | Women who delivered normal infants at term without labor via elective cesarean section | PE 23 (20-26), controls 30 (21-38) ^#^ | PE 2/7 nulliparous, controls 1/7 ^#^ | PE 35 (31-39), controls 38 (37-39) ^#^ | Hypertension (systolic ≥ 140 mm Hg, or diastolic ≥90 mmHg, on at least 2 occasions, at least 4 hours apart, and proteinuria of ≥300 mg in a 24-hour urine collection or urine dipstick measurement of ≥ 2+ | 7 | 6 |
| Zhu 2009 | China | Women with severe PE who delivered by elective cesarean section in the absence of labor, without any other maternal complications, or birthweight <10^th^ percentile | Women with normal pregnancies (matched for gestational age at delivery) who delivered by elective cesarean section in the absence of labor | PE 31 (3.8), controls 31 (3.7)$ | All nulliparous | PE 35 (2.7), controls 37 (2.2) $ | Severe PE: either severe hypertension (systolic blood pressure of ≥160 mm Hg and/or diastolic blood pressure of ≥110 mm Hg on at least 2 occasions 6 hours apart) plus mild proteinuria or mild hypertension plus severe proteinuria (>2 g/24 hr or >2+ by dipstick) | 8 | 8 |

†: these data are reported for a larger group of women (cases n=24 and controls n=26) that includes the women in the microarray experiments.

#: these data are reported for a larger group of women in the control group (n=7) that includes the women in the microarray experiments.

^$^: these data are reported for a larger group of women (cases n=15 and controls n=11) that includes the women in the microarray experiments.
