# Supplementary material for: Differentially Expressed Genes in the Pre-Eclamptic Placenta: A Systematic Review and Meta-Analysis
Source: PLoS One. 2013 Jul 12;8(7):e68991. doi: 10.1371/journal.pone.0068991 (PMC3709893; doi:10.1371/journal.pone.0068991)
Supplement: Table S2 — Experimental data: details of the sampling, testing and analysis processes of all studies, as they were reported in the original papers. ANOVA: analysis of variance; PE: pre-eclampsia. a: A complete list is defined as the complete list of genes identified as differentially expressed according to the studies’ selection criteria. (DOCX) [file pone.0068991.s002.docx]

**Table S2. Experimental data: details of the sampling, testing and analysis processes of all studies, as they were reported in the original papers**

**Part 1: Studies on third-trimester placental tissue RNA expression**

| **First author,**  **year** | **Tissue sampling** | **Sample storage** | **RNA isolation** | **Pooled samples** | **Platform** | **Normalization method** | **Selection**  **criteria** | **Complete list ^a^: # genes identified and reported** | **q-PCR validation** |
| --- | --- | --- | --- | --- | --- | --- | --- | --- | --- |
| Ahr 2006 | Placenta | Stored at -70°C | RNeasy (Qiagen, Hilden) | Not reported | Hu19k2 (University Health Network, Ontario Cancer Institute, Ontario, Canada) | Median centered | >5 fold change | No: 31/200 identified genes were reported | Yes (results not shown in paper) |
| Centlow 2011 | Villous tissue from one cotyledon in central portion of the placenta | Stored at −80°C | TRIzol (Invitrogen, Carlsbad, USA) | No | Microarrays produced at Swegene DNA Microarray Resource Center, Department of Oncology, Lund University, Sweden. Probes obtained from Operon (Operon Biotechnologies, Germany) | Lowess normalization and then corrected for common reference bias using gene median centering | ≥1.5 fold change and false discovery rate modified t-test with p-value <0.005 | Yes: 46 genes | Yes, for INHA and TGFβ1 |
| Enquobahrie 2008 | A medial and lateral sample from each placental quadrant from maternal side that consisted primarily of the villous tissue, uteroplacental arteries, and some decidua basalis | RNAlater (Qiagen Inc, Valencia, CA) | RNeasy Fibrous Tissue Midi (Qiagen, Inc) | No | Human Genome Array Ready Oligo Set (version 2.1; Operon Biotechnologies Inc, Huntsville, AL) | Lowess algorithm | Student's t-test (2 sample, unequal variances) and ≥1.5 fold-change and false discovery rate ≤10% in SAM analysis | No: 49/58 identified genes were reported | Yes, for LEP, FLT1, CYP11A, F2R, FCGR2B, CDO1, PROCR, TPBG and NR4A2 |
| Gack 2005 | Ten- to 20-mm-thick sections of placental villi from two distinct locations between the chorionic and basal plates | RNAlater (Ambion) | Trizol (Invitrogen, Germany) | No | Nonredundant, sequence-validated human complementary DNAs (RZPD set, Berlin) spotted on poly-L-lysin–coated slides (Sigma-Aldrich, Munich, Germany) | Variance stabilization | Wilcoxon rank-sum test p-value ≤0.05 and ≥2.5- fold change | Yes: 91 genes | Yes, for ADAM12-S, EB13, LIM and MIFR-2 |
| Han 2006 | Five interface specimens between the maternal and fetal surfaces of each placenta | Liquid nitrogen at -70°C | Not reported | Yes | Atlas Human apoptosis array (Clontech Laboratories, Inc., USA) | Not reported | Independent Student’s t-test and >2 fold change | Yes: 3 genes | Yes, of all 3 identified genes |
| Hansson 2006 | One 10 mm^3^ cube consisting mainly of villi from the central part of the placenta outside areas with macroscopic evidence for necrosis or infarctions | Stored at −80°C | Trizol (Invitrogen) | Not reported | Human cDNA microarrays were printed on poly-L-lysine coated glass slides using an OmniGrid arrayer (GeneMachines, San Carlo, CA) | Cross-array normalization and ratio averaging | Distance-based method with discriminative weight p-value 0.0001 derived from 10000 random permutations and average fold change >1.6 | No: 66/80 identified genes were reported | Yes, for ACP5, Calm2 and RELA |
| Heikkilä 2005 | Two representative samples with the whole thickness of the placenta halfway between margin of the placenta towards the origin of the umbilical cord in a healthy looking area | Stored at -70°C | Trizol method (Gibco BRL, Eggenstein, Germany) | No (results were pooled in the analyses) | LifeGrid 1.1 filters (IncyteGenomics, Wilmington, Delaware, USA) | Normalized by the average intensities of the filters | 2 fold change and p-value 0.05 and signal intensity ≥1000 * background | No: 61 of approximately 437 identified genes/cDNAs were reported | Yes, for I2, KCNJ5, eNOS3, ANXA3, Egr-1, HK2, PCSK7, PP2, TCEB2 , SP140 and GAPDH |

**Part 1 (continued)**

| **First author,**  **year** | **Tissue sampling** | **Sample storage** | **RNA isolation** | **Pooled samples** | **Platform** | **Normalization method** | **Selection**  **criteria** | **Complete list ^a^: # genes identified and reported** | **q-PCR validation** |
| --- | --- | --- | --- | --- | --- | --- | --- | --- | --- |
| Herse 2007 | Placenta biopsies | Liquid nitrogen and stored at -80°C | RNeasy mini kit (Qiagen) | Not reported | Chip U133 plus 2 (Affymetrix) | Not reported | ≥4-fold change in means from triplicates and p-value 0.05 in t-test or Mann-Whitney test | No: 10 identified were reported, total of identified genes not reported | Yes, for 10 genes not reported among identified genes |
| Hoegh 2010 | Placental biopsies from the maternal side of approximately 0.2–0.5 cm^3^ from the center of the cotyledons evenly across each placenta | RNAlater solution (Ambion, Austin, Texas) and freezing at -80°C or liquid nitrogen stored at -80°C | Trizol (Invitrogen, Paisley, UK) and clean-up using RNeasy Mini Kit (QIAGEN, GmbH, Hilden, Germany) | Yes: into a total of 6 groups; 3 groups each consisting of RNA from 3 pre-eclamptic placentas and 3 groups each consisting of RNA from 3 controls | GeneChip HG-U133A (Affymetrix Inc., Santa Clara, CA, USA) | Multiarray invariant-set normalization | ≥1.2 fold change and ≥ 50 units absolute change in signal intensity and p-value ≤ 0.05 | Yes: 21 genes | Yes, for Inhibin, Leptin, Fibulin 1A and Tyrosine 3-monooxygenase/tryptophan 5-monooxygenase activation protein |
| Järvenpää 2007 | Placenta, not otherwise reported | Frozen (not otherwise reported) | PAXgene blood RNA kit (Qiagen GmbH) | No | Affymetrix Human Genome U133 Plus 2 arrays | Invariant Set Normalization | Two-samples t-test and >1.5 fold change | No: only 13 angiogenesis-associated genes were reported | Yes, for JAG1, COL18A1 and FLT1 |
| Järvenpää 2009 | Biopsies from the fetal side of the placenta, about 2 cm from the insertion of the cord | Liquid nitrogen | PAXgene blood RNA kit (Qiagen GmbH, Hilden, Germany) | No | Affymetrix Human Genome U133 Plus 2 arrays | Not reported | Not reported | No, only 2 adiponectin- and leptin-related genes were reported | Yes, for leptin, ADIPOR1 and ADIPOR2 |
| Kang 2011 | Chorionic tissue dissected from full-thickness, macroscopically normal areas near the umbilical cord insertion to the periphery including the basal and chorionic plates. The maternal deciduas and amniotic membranes were removed | Liquid nitrogen | TRIzol reagent (Invitrogen, USA) and additional RNA clean-up using an RNeasy Mini Kit (Qiagen Inc., Germany) | Not reported | Codelink Human Whole Genome Bioarrays (GE Healthcare, USA) | Default settings in Codelink Expression Analysis v4.0 software | Student’s t-test, Welch’s t-test, and/or Wilcoxon Mann–Whitney test, ≥2 fold change and < 0.01 false discovery rate | Yes: 79 genes | Yes, for FLT1, ITGA5, SIGLEC6, LEP, EB13 and HTRA1 |

**Part 1 (continued)**

| **First author,**  **year** | **Tissue sampling** | **Sample storage** | **RNA isolation** | **Pooled samples** | **Platform** | **Normalization method** | **Selection**  **criteria** | **Complete list ^a^: # genes identified and reported** | **q-PCR validation** |
| --- | --- | --- | --- | --- | --- | --- | --- | --- | --- |
| Lee 2010 | A central area of placental tissue, maternal deciduas and amniotic membranes were removed | Liquid nitrogen | RiboPure kit (Ambion, Austin, TX, USA) and further purified with RNeasy Mini Kit (Qiagen, Valencia, CA,USA) | Yes: equal amounts of total RNA were pooled from the PE and control groups and reverse transcribed per group | Agilent Human 4X44K Oligomicroarrays chip (Agilent Technology, Santa Clara, CA, USA) | Intensity-dependent normalization | Student’s t-test p-value <0.05 and ≥2 fold change | No: 11/415 identified genes were reported | Yes, for OSM, FLT1, VEGFA, SMOX, CYP26A1 and LDH |
| Liu 2008 | Four or five placental lobes | NA (samples not stored) | Trizol (Invitrogen, Carlsbad, CA, USA) and purified using NucleoSpin RNA clean-up kit (Macherey- Nagel, Düren, Germany) | Not reported | CapitalBio 22 K Human Genome Oligo Array, (Beijing, China) | Intensity-dependent lowess program | Student’s t-test p-value ≤0.05 | Yes: 10 genes | Yes, for HSP70 |
| Mayor-Lynn 2011 | The overlaying fetal membranes were removed and several small pieces of placental villous tissues were randomly collected from different areas | RNAlater (Qiagen Inc, Valencia, California) and stored in liquid nitrogen | Trizol (Invitrogen, Carlsbad, California) | Not reported | HumanRef-12 v3 Expression BeadChip (Illumina, Inc, San Diego, California) | Background subtracted and globally normalized and probes with differential score of ≤13 were independently removed from each cohort | P-value ≤.05 (ANOVA, Tukey test) and 2-fold change | Yes: 120 genes | Yes, for MMP-1, MMP-9, TIMP-3, ADAM-17, ADAM-30, STC2, CRH, CRHBP, SOCS1 and END2 |
| Nishizawa 2011 | 1 cm sections of central chorionic villi from four different central areas between the basal and chorionic plates. Maternal deciduas and amnionic membranes were removed | Liquid nitrogen | RNeasy mini-kit (Qiagen Inc., Valencia, CA) | No | Affymetrix Human Exon 1.0 ST array (Affymetrix, Santa Clara, CA) | Per chip and per gene quantile normalization | ANOVA p-value <0.05 and ≥ 1.5 fold change | No: 91/245 identified genes were reported | Yes, for TP53, BCL6, ENG< ADFP, APLP1, SURF1, BAX, FASLG en P53AIP1 |
| Nishizawa 2007 | 1-cm-thick sections of placental villi from the central area of chorionic tissue between basal and chorionic plates. The maternal deciduas and amnionic membranes were removed | Liquid nitrogen | RNeasy mini-kit (Qiagen Inc., Valencia, CA) | Not reported | Whole Human Genome Oligo Microarray Kit (Agilent Technologies) | Intensity dependent normalization (Lowess) and corrected for median background intensity | Student’s t-test p-value <0.05 and ≥ 1.5 fold change and average normal expression level ≥500 | No: 41/137 identified genes were reported | Yes, for LEP, FLT1, INHBA, MIG6, EB13, ADFP, CCL3, and PLA2R1 |
| Pang 2003 Br J Biomed Sci | 1 cm^3^ chorionic tissue blocks cut from the same position in the maternal face of placentas | Liquid nitrogen | Trizol and purified with Oligotex mRNA Kit (Qiagen Inc. (Valencia, CA, USA) | Yes, separately for PE and controls | Commercially available microarrays (Takara Shuzo, Otsu, Shiga, Japan) | Background-subtracted and normalized according to the housekeeping genes | 2 fold change | No: 27 identified genes were reported, total number of identified genes not reported | Not reported |

**Part 1 (continued)**

| **First author,**  **year** | **Tissue sampling** | **Sample storage** | **RNA isolation** | **Pooled samples** | **Platform** | **Normalization method** | **Selection**  **criteria** | **Complete list ^a^: # genes identified and reported** | **q-PCR validation** |
| --- | --- | --- | --- | --- | --- | --- | --- | --- | --- |
| Pang 2003  J Perinat Med | 1 cm^3^ chorionic tissue blocks cut from the same position in the maternal face of placentas | Liquid nitrogen | Trizol and purified with Oligotex mRNA Kit (Qiagen Inc. (Valencia, CA, USA) | Not reported | IntelliGene^TM^ Human Cytokine CHIP (Ver.1.1) (Takara Shuzo Co., Ltd., Biomedical Group, Otsu, Shiga, Japan) | Background- subtracted and normalized according to the housekeeping genes | 2 fold change | No: 83/162 identified genes were reported | Yes (results not shown in paper) |
| Pang 2004  J Perinat Med | 1 cm^3^ chorionic tissue blocks cut from the same position in the maternal face of placentas | Liquid nitrogen | Trizol and purified with Oligotex mRNA Kit (Qiagen Inc. (Valencia, CA, USA) | Not reported | IntelliGene Human Cytokine CHIP (Ver.1.1) and IntelliGene Human DNA CHIP for Endocrine Disruption Study (Ver.1.1) ( Takara Shuzo Co., Ltd., Otsu, Shiga, Japan) | Background- subtracted and normalized according to the housekeeping genes | 2 fold change | No: 43/approximately 129 identified genes were reported | Yes (results not shown in paper) |
| Pang 2004 Arch Gynecol Obstet | 1 cm^3^ chorionic tissue blocks cut from the same position in the maternal face of placentas | Liquid nitrogen | Trizol and purified with Oligotex mRNA Kit (Qiagen Inc. (Valencia, CA, USA) | Not reported | IntelliGene™ Human DNA CHIP for Endocrine Disruption Study (Ver.1.1) (Takara Shuzo Co., Ltd., Otsu, Shiga, Japan) | Background- subtracted and normalized according to the housekeeping genes | 2 fold change | No: 34 identified genes were reported, total number of identified genes unclear | Not reported |
| Reimer 2002 | A defined central chorionic tissue area was dissected and the maternal decidua and amniotic membrane were removed | Liquid nitrogen | RNeasy mini-kit (Qiagen Inc., Valencia, CA, USA) | Yes: samples from each group (PE and controls) were pooled | HuGeneFL array (Affymetrix) | Normalized to the housekeeping gene glyceraldehydes-3-phosphate dehydrogenase (GADPH) mRNA concentration | 3 fold change | Yes: 56 genes | Yes, for leptin and integrin α1 |
| Sitras 2009 | Chorionic tissue from approximately 2 cm^3^ beside the umbilical cord insertion, from the middle layer of placenta midway between maternal and fetal surfaces from macroscopically normal areas excluding sites of infarction, haemorrhage and fibrin deposition | RNAlater solution (RNA stabilization reagent, Qiagen GmbH, Germany) and stored at -70 °C | MagNa Pure Compact RNA isolation kit and the MagNa Pure Compact Instrument (Roche Applied Science, Germany) | No | The Human Genome Survey Microarray v.2.0 (Applied Biosystems) | Quantile normalization | 2 fold change and ANOVA p-value <0.01 | Yes: 186 genes | Yes, for PPIA, LEP, FLJ90650, FLT1, PDGFD, COX17, BRSK2, LHB, INHA, CYP1B1, PAPPA2, COL17A1, CRLF1, PLA2G4A, ADCY2, PTGS2, METT5D1 |
| Soleymanlou 2005 | Multiple specimens from central and peripheral regions and both the maternal and fetal sides, outside areas with calcified, necrotic, or visually ischemic tissue | Not reported | Rneasy kit (QIAGEN, Santa Clarita, CA) | Yes: samples from the same experimental conditions were pooled | 1.7k version 4 human microarrays | Not reported | Student’s two-way t-test p-value <0.05 | Yes: 34 genes | Yes, for VEGF and integrin α6 |

**Part 1 (continued)**

| **First author,**  **year** | **Tissue sampling** | **Sample storage** | **RNA isolation** | **Pooled samples** | **Platform** | **Normalization method** | **Selection**  **criteria** | **Complete list ^a^: # genes identified and reported** | **q-PCR validation** |
| --- | --- | --- | --- | --- | --- | --- | --- | --- | --- |
| Tsai 2011 | 2 cm^3^ within 5 cm of the placental umbilical insertion site excluding fetal membranes | Liquid nitrogen | Totally RNA kit (Ambion) | Yes: samples were hybridized in two batches | Illumina Human6-v2 BeadArrays | Quantile normalized | ANOVA with Benjamini-Hochberg false discovery rate <0.05 and Bonferroni post-hoc test p-value <0.05 | Yes: 128 genes | Yes, for SIAE, ENG, PIK3R1, RHOG, CD4, CXCR4 and ENG |
| Tsoi 2003 | Placental villi from between the chorionic and basal plates | Liquid nitrogen | TRIzol Reagent (Life Technologies, Rockville, MD) | Yes: samples from each group (PE and controls) were pooled | Two GeneFilter microarrays (GF211) (ResGen, Huntsville, AL) | Background subtracted and average total intensity normalization | t-test and >1.6 fold change and average expression level >4000 units | Yes: 20 genes | No (validated for GP-M and β-actin with northern blot) |
| Vaiman 2005 | Villous tissue between the decidual and chorionic plates, free of fetal membranes, vessels and tissue from maternal origin, at six to ten various locations per placenta | Trizol™ reagent (Life Technologies, Cergy, France) and stored at -80°C | Trizol™ reagent (Life Technologies, Cergy, France) | No | Two reciprocal suppressive/subtractive hybridization procedures (SSH) were applied to early (11 weeks) human placental villi after incubation either in normoxic or in hypoxic conditions. The clones from both libraries were double-spotted on nylon membranes (Hybond N+, Amersham) | By reference to a maximum signal intensity fixed at 120 (arbitrary units) | Distribution of gene expression tested with Chi^2^ analysis | No: 94 identified genes were reported, total number of identified genes not reported | Not reported |
| Várkonyi 2011 | Villous tissue samples from central cotyledons close to the umbilical cord | Stored at -80°C | RNeasy Fibrous Tissue Mini Kit (Qiagen, Germany), purified with RNeasy Mini Kit (Qiagen) | No | Agilent 44K Whole Human Genome Oligo Microarray Chips | Background corrected and quantile normalized | Moderated t-test with false discovery rate adjusted p-value <0.25 and fold change ≥2 | No, 20/350 identified genes were reported | Yes, for 11 genes including LEP, CGB, LHB, SICLEC6, ARHGEF4 and MGST1 |
| Winn 2009 | Basal plate specimens of the maternal fetal interface | Snap-frozen | TRIzol | No | High-density HG-U133A and HGU133B GeneChips (Affymetrix, Santa Clara, CA) | Background subtraction, quantile normalization, and probe set summarization, then global median normalization at the probe set level | LOD (Log Odds of Differential expression) ratios with B>0 | Yes: 55 genes | Yes, for CRH, Leptin, FLT-1, SIGLEC6, PAPP-A2, ESP8L1, KIAA1211, ASB2 and HSD17B1 |

**Part 1 (continued)**

| **First author,**  **year** | **Tissue sampling** | **Sample storage** | **RNA isolation** | **Pooled samples** | **Platform** | **Normalization method** | **Selection**  **criteria** | **Complete list ^a^: # genes identified and reported** | **q-PCR validation** |
| --- | --- | --- | --- | --- | --- | --- | --- | --- | --- |
| Zhou 2006 | Central chorionic tissue around 0.5 x 0.5 x 0.5 cm, the maternal deciduas and amnionic membrane were removed | RNAlater (Qiagen, Valencia, Calif., USA) | TRIzol Reagent (Invitrogen, Gaithersburg, Md., USA) and further purified with RNeasy mini kit (Qiagen) | Yes: control samples were pooled | Microarray including well-characterized Homo sapiens genes (CapitalBio) | Space and intensity-dependent normalization based on lowess program | ≥1.5 fold change | Yes: 85 genes | Yes, for 20 genes including LPL, ADM, SOD1, PLAU and HLA-DRA |

**Part 2: Studies on third-trimester placental tissue microRNA expression**

| **First author,**  **year** | **Tissue sampling** | **Sample storage** | **RNA isolation** | **Pooled samples** | **Platform** | **Normalization method** | **Selection**  **criteria** | **Complete list ^a^: # miRNAs identified and reported** | **q-PCR validation** |
| --- | --- | --- | --- | --- | --- | --- | --- | --- | --- |
| Enquobahrie 2011 | A medial and lateral sample from each placental quadrant from maternal side that consisted primarily of the villous tissue, uteroplacental arteries, and some decidua basalis | RNAlater (Qiagen Inc, Valencia, CA) | RNASTAT60 (Tel-Test, Friendswood, TX) and RNeasy MinElute (Qiagen Inc) | Not reported | Human mature microRNA microarray (Microarrays Inc., Huntsville, AL) | Lowess algorithm | Absolute fold change >1.5 and ANOVA with Benjamini-Hochberg false discovery rate adjusted p-value <0.05 | Yes: 8 miRNAs | Yes, for miR-518c, miR-1, miR-103, miR-15a, miR-584, miR-324-5p, miR-200b, miR-210 and miR-154 |
| Hu 2009 | Chorionic tissue blocks (~1 cm^3^) from the central part of the placenta only. Contamination with decidua and amniotic membranes was excluded by morphological observation | Liquid nitrogen and stored at -80°C | Trizol Reagent (Invitrogen, Carlsbad, CA, USA) | Yes: 4 normal placentas were pooled to form a control group | CapitalBio Mammalian miRNA Array V3.0 (CapitalBio Corp, Beijing, China) | Not reported | Student’s t-test p-value <0.05 and 2 fold change | Yes: 27 miRNAs | Yes, for miR-181a, miR-195, miR-222, miR-16, miR-29b, miR-26b and miR-335 |
| Mayor-Lynn 2010 | The overlaying fetal membranes were removed and several small pieces of placental villous tissues were randomly collected from different areas | RNAlater (Qiagen Inc, Valencia, California) and stored in liquid nitrogen | Trizol (Invitrogen, Carlsbad, California) | Not reported | Custom-developed microarray by Ocean Ridge Biosciences (Jupiter, Florida) | The mean triplicate spot intensity values for each probe was determined, subtracted from the median background values and normalized relative to control miRNA (Ambion) added to each sample | ANOVA with Tukey post-hoc test and 1.5-fold change cutoff | Yes: 78 miRNA’s | Yes, for miR-15b, miR-181a, miR-200C, miR-210, miR-296, miR-483-5p and miR-493 |
| Zhu 2009 | Tissue blocks (~1 cm^3^ each) randomly sampled from different lobules (10 sites) dissected from the decidual side of the placentas avoiding macroscopic areas of necrosis and infarction | Liquid nitrogen and stored at -80°C | Trizol reagent (Invitrogen, Carlsbad, CA) | No | miRNA microarray chip (Exiqon) | Median centered and global median normalization | P-value <0.05 and 1.8 fold change | Yes: 34 miRNAs | Yes, for miR-210, miR-152, miR-411, miR-377, miR-518b, miR-81a, miR-363 and miR-542-3p |
