# Supplementary material for: Differentially Expressed Genes in the Pre-Eclamptic Placenta: A Systematic Review and Meta-Analysis
Source: PLoS One. 2013 Jul 12;8(7):e68991. doi: 10.1371/journal.pone.0068991 (PMC3709893; doi:10.1371/journal.pone.0068991)
Supplement: Table S3 — Meta signature of third-trimester placental tissue mRNA expression. Genes present in 3 or more complete signatures, or 3 or more signatures in total, are part of the meta-signature. Genes incorporated in the meta-signature are ordered by (1) the total number of publications that reported the gene as differentially expressed and (2) alphabetically. References printed in bold indicate publications that reported a complete signature. Enquobahrie 2008, Kang 2011, Pang (combined) and Vaiman 2005 did not report the direction of differential expression (upregulation or downregulation). For comparison, the number of times a gene is present in complete signatures below the significance threshold is shown in grey. *: Results were inconsistent among studies, the majority of studies reported the result presented here. –: No consensus among studies: equal numbers of studies reported upregulated expression and downregulated expression in pre-eclampsia. 1st trimester expression was scored using microarray data from control chorionic villous sampling (NCBI Gene Expression Omnibus GSE12767) and 1st trimester placenta samples (NCBI Gene Expression Omnibus GSE9984). Microarrays were analyzed for expression using The Gene Expression Barcode 2.0 (http://barcode.luhs.org/). ‘+’ indicates that in >90% of the samples expression of the gene was detected, ‘–’ indicates that in <10% of the samples expression of the gene was detected. The remaining samples showed a variable degree of expression (‘+/−’). (DOCX) [file pone.0068991.s003.docx]

**Table S3. Meta signature of third-trimester placental tissue mRNA expression**

| **Gene symbol** | **Gene name** | **Chromosomal location** | **Encodes extracellular protein** | **1^st^ trimester expression** | **Number of times present in complete signature** | **Number of times present in any signature** | **Expression in pre-eclampsia** | **References** |
| --- | --- | --- | --- | --- | --- | --- | --- | --- |
| LEP | leptin | 7q31.3 | + | + | 5 | 12 | Up | **Hoegh 2010, Kang 2011, Reimer 2002, Sitras 2009, Winn 2009,** Enquobahrie 2008, Herse 2007, Järvenpää (combined), Lee 2010, Nishizawa 2007, Nishizawa 2011, Varkonyi 2011 |
| FLT1 | fms-related tyrosine kinase 1 | 13q12 | + | +/- | 4 | 11 | Up | **Centlow 2011, Kang 2011, Sitras 2009, Winn 2009,** Enquobahrie 2008, Herse 2007, Järvenpää (combined), Lee 2010, Nishizawa 2007, Nishizawa 2011, Vaiman 2005 |
| INHBA | inhibin, beta A | 7p15-p13 | + | +/- | 5 | 9 | Up | **Gack 2005, Hoegh 2010, Reimer 2002, Sitras 2009, Winn 2009,** Ahr 2006, Nishizawa 2007, Nishizawa 2011, Pang (combined) |
| ENG | endoglin | 9q34.11 | + | +/- | 5 | 8 | Up | **Gack 2005, Kang 2011, Sitras 2009, Tsai 2011, Winn 2009,** Nishizawa 2007, Nishizawa 2011, Pang (combined) |
| EBI3 | Epstein-Barr virus induced 3 | 19p13.3 | + | + | 3 | 6 | Up* | **Gack 2005, Kang 2011, Mayor-Lynn 2011,** Enquobahrie 2008, Nishizawa 2007, Nishizawa 2011 |
| INHA | inhibin, alpha | 2q33-q36 | + | +/- | 4 | 6 | Up | **Centlow 2011, Reimer 2002, Sitras 2009, Winn 2009,** Enquobahrie 2008, Nishizawa 2011 |
| SIGLEC6 | sialic acid binding Ig-like lectin 6 | 19q13.3 | + | + | 3 | 6 | Up | **Kang 2011, Sitras 2009, Winn 2009,** Enquobahrie 2008, Vaiman 2005, Varkonyi 2011 |

**Table S3 (continued)**

| **Gene symbol** | **Gene name** | **Chromosomal location** | **Encodes extracellular protein** | **1^st^ trimester expression** | **Number of times present in complete signature** | **Number of times present in any signature** | **Expression in pre-eclampsia** | **References** |
| --- | --- | --- | --- | --- | --- | --- | --- | --- |
| BCL6 | B-cell CLL/lymphoma 6 | 3q27 |  | +/- | 3 | 5 | Up | **Reimer 2002, Sitras 2009, Winn 2009,** Enquobahrie 2008, Nishizawa 2011 |
| CGB | chorionic gonadotropin, beta polypeptide | 19q13.32 | + | + | 2 | 5 | Up | **Sitras 2009, Winn 2009,** Heikkila 2005, Nishizawa 2011, Varkonyi 2011 |
| CRH | corticotropin releasing hormone | 8q13 | + | +/- | 2 | 5 | Up* | **Mayor-Lynn 2011, Winn 2009,** Heikkila 2005, Nishizawa 2007, Nishizawa 2011 |
| HTRA1 | HtrA serine peptidase 1 | 10q26.3 | + | + | 3 | 5 | Up | **Kang 2011, Sitras 2009, Winn 2009,** Heikkila 2005, Nishizawa 2011 |
| PAPPA2 | pappalysin 2 | 1q23-q25 | + | +/- | 3 | 5 | Up* | **Mayor-Lynn 2011 2011, Tsai 2011, Winn 2009,** Nishizawa 2011, Varkonyi 2011 |
| CYP11A1 | cytochrome P450, family 11, subfamily A, polypeptide 1 | 15q23-q24 |  | + | 3 | 4 | Up* | **Gack 2005, Kang 2011, Mayor-Lynn 2011,** Enquobahrie 2008 |
| FSTL3 | follistatin-like 3 | 19p13 | + | +/- | 1 | 4 | Up | **Sitras 2009,** Enquobahrie 2008, Herse 2007, Nishizawa 2011 |
| KRT19 | keratin 19 | 17q21.2 |  | +/- | 2 | 4 | Up | **Gack 2005, Sitras 2009,** Nishizawa 2011, Vaiman 2005 |

**Table S3 (continued)**

| **Gene symbol** | **Gene name** | **Chromosomal location** | **Encodes extracellular protein** | **1^st^ trimester expression** | **Number of times present in complete signature** | **Number of times present in any signature** | **Expression in pre-eclampsia** | **References** |
| --- | --- | --- | --- | --- | --- | --- | --- | --- |
| SLCO2A1 | solute carrier organic anion transporter family, member 2A1 | 3q21 |  | +/- | 2 | 4 | Up | **Kang 2011, Winn 2009,** Enquobahrie 2008, Nishizawa 2011 |
| SOD1 | superoxide dismutase 1 | 21q22.11 | + | + | 3 | 4 | – | **Centlow 2011, Gack 2005, Zhou 2006,** Pang (combined) |
| AQP1 | aquaporin 1 | 7p14 |  | +/- | 2 | 3 | Up* | **Reimer 2002, Tsoi 2003,** Nishizawa 2011 |
| BHLHE40 | basic helix-loop-helix family, member e40 | 3p26 |  | +/- | 1 | 3 | Up | **Sitras 2009,** Nishizawa 2007, Nishizawa 2011 |
| CGA | glycoprotein hormones, alpha polypeptide | 6q12-q21 | + | + | 1 | 3 | Up | **Tsoi 2003,** Heikkila 2005, Vaiman 2005 |
| EZR | ezrin | 6q25.3 |  | +/- | 1 | 3 | Up | **Gack 2005,** Vaiman 2005, Pang (combined) |
| F5 | coagulation factor V | 1q23 | + | +/- | 2 | 3 | Down | **Mayor-Lynn 2011, Winn 2009,** Nishizawa 2011 |
| HEXB | hexosaminidase B | 5q13 |  | + | 1 | 3 | Up | **Tsai 2011,** Enquobahrie 2008, Nishizawa 2007 |
| HSD17B1 | hydroxysteroid (17-beta) dehydrogenase 1 | 17q11-q21 |  | +/- | 2 | 3 | Down | **Centlow 2011, Winn 2009,** Nishizawa 2011 |
| HTRA4 | HtrA serine peptidase 4 | 8p11.22 | + | + | 1 | 3 | Up | **Sitras 2009,** Nishizawa 2011, Varkonyi 2011 |

**Table S3 (continued)**

| **Gene symbol** | **Gene name** | **Chromosomal location** | **Encodes extracellular protein** | **1^st^ trimester expression** | **Number of times present in complete signature** | **Number of times present in any signature** | **Expression in pre-eclampsia** | **References** |
| --- | --- | --- | --- | --- | --- | --- | --- | --- |
| IGFBP1 | insulin-like growth factor binding protein 1 | 7p13-p12 | + | +/- | 1 | 3 | Up* | **Gack 2005,** Ahr 2006, Pang (combined) |
| LHB | luteinizing hormone beta polypeptide | 19q13.32 | + | +/- | 2 | 3 | Up | **Sitras 2009, Winn 2009,** Varkonyi 2011 |
| PGF | placental growth factor | 14q24.3 | + | + | 2 | 3 | Up* | **Gack 2005, Zhou 2006,** Pang (combined) |
| PHYHIP | phytanoyl-CoA 2-hydroxylase interacting protein | 8p21.3 |  | - | 3 | 3 | Up | **Reimer 2002, Sitras 2009, Winn 2009** |
| PLEC | plectin | 8q24 |  | +/- | 3 | 3 | Up | **Gack 2005, Sitras 2009, Zhou 2006** |
| PVRL4 | poliovirus receptor-related 4 | 1q22-q23.2 | + | - | 1 | 3 | Up | **Sitras 2009,** Nishizawa 2007, Nishizawa 2011 |
| RDH13 | retinol dehydrogenase 13 (all-trans/9-cis) | 19q13.42 |  | +/- | 3 | 3 | Up | **Sitras 2009, Tsai 2011, Winn 2009** |
| SASH1 | SAM and SH3 domain containing 1 | 6q24.3 |  | +/- | 3 | 3 | Up | **Hoegh 2010, Sitras 2009, Winn 2009** |
| SEMA4C | sema domain, immunoglobulin domain (Ig), transmembrane domain (TM) and short cytoplasmic domain, (semaphorin) 4C | 2q11.2 |  | +/- | 2 | 3 | Up | **Sitras 2009, Tsai 2011,** Vaiman 2005 |

**Table S3 (continued)**

| **Gene symbol** | **Gene name** | **Chromosomal location** | **Encodes extracellular protein** | **1^st^ trimester expression** | **Number of times present in complete signature** | **Number of times present in any signature** | **Expression in pre-eclampsia** | **References** |
| --- | --- | --- | --- | --- | --- | --- | --- | --- |
| SPAG4 | sperm associated antigen 4 | 20q11.21 |  | - | 3 | 3 | Up | **Sitras 2009, Tsai 2011, Winn 2009** |
| SPP1 | secreted phosphoprotein 1 | 4q22.1 | + | +/- | 2 | 3 | Up | **Gack 2005, Tsoi 2003,** Pang (combined) |
| TNFSF10 | tumor necrosis factor (ligand) superfamily, member 10 | 3q26 | + | +/- | 0 | 3 | Up | Hansson 2006, Heikkila 2005, Pang (combined) |
| TREM1 | triggering receptor expressed on myeloid cells 1 | 6p21.1 | + | +/- | 1 | 3 | Up | **Sitras 2009,** Nishizawa 2011, Varkonyi 2011 |
| VEGFA | vascular endothelial growth factor A | 6p12 | + | +/- | 1 | 3 | Up* | **Soleymanlou 2005,** Järvenpää (combined), Lee 2010 |
| VIM | vimentin | 10p13 |  | +/- | 1 | 3 | – | **Gack 2005,** Vaiman 2005, Pang (combined) |
