# Supplementary material for: Differentially Expressed Genes in the Pre-Eclamptic Placenta: A Systematic Review and Meta-Analysis
Source: PLoS One. 2013 Jul 12;8(7):e68991. doi: 10.1371/journal.pone.0068991 (PMC3709893; doi:10.1371/journal.pone.0068991)
Supplement: Document S1 — Electronic search strategy PubMed. (DOC) [file pone.0068991.s004.doc]

(Placenta[mh] OR Placental Circulation[mh] OR Maternal-Fetal Exchange[mh] OR placent*[tiab] OR placenta[jour] OR Uteroplacent*[tiab] OR Fetoplacent*[tiab] OR foetoplacent*[tiab] OR trophoblast*[tiab] OR Cytotrophoblast*[tiab] OR Syncytiotrophoblast*[tiab] OR extravill*[tiab] OR decidu*[tiab] OR chorioamn*[tiab] OR feto-maternal[tiab]) AND (Gene Expression Profiling[mh] OR Microarray Analysis[mh:noexp] OR Oligonucleotide Array Sequence Analysis[mh] OR microarray*[tiab] OR micro-array*[tiab] OR gene expression profil*[tiab] OR gene expression analys*[tiab] OR Gene Expression Signature*[tiab] OR gene expression data[tiab] OR gene expression array*[tiab] OR transcriptom*[tiab] OR transcript profil*[tiab] OR affymetrix[tiab] OR cDNA array*[tiab] OR RNA array* OR DNA array*[tiab] OR mRNA array*[tiab] OR Oligonucleotide Array*[tiab] OR array-CGH*[tiab] OR micro-chip*[tiab] OR microchip*[tiab] OR DNA Chip*[tiab] OR Gene Chip*[tiab] OR genechip*[tiab])
