# Supplementary material for: Differentially Expressed Genes in the Pre-Eclamptic Placenta: A Systematic Review and Meta-Analysis
Source: PLoS One. 2013 Jul 12;8(7):e68991. doi: 10.1371/journal.pone.0068991 (PMC3709893; doi:10.1371/journal.pone.0068991)
Supplement: Document S2 — Studies that were excluded after screening of full text and new studies that were included, as described in Figure 1 . (DOC) [file pone.0068991.s005.doc]

Excluded studies

- No report on placental tissue but other tissue (*n*=6)
  - Chorionic villous tissue [1,2]
  - Decidua [3–6]
- Control group not eligible (*n*=1)
- Control group composed of pregnancies complicated by pre-eclampsia and small for gestational age fetuses [7]
- Report on specifically selected (number of) gene(s) only (*n*=4)
  - Only ceruloplasmin [8]
  - Only HLA-G [9]
  - Only STOX1 [10]
  - Only ROCK2 [11]
- No report on individual genes, only functional clusters (*n*=1) [12]
- Duplicate or subgroup analyses of other publication (*n*=2)
  - Report on same genes as in included paper from Järvenpää *et al.* 2007 [13]
  - Report on same sample of pre-eclamptic and healthy women as in included paper from Centlow *et al.* 2011, and two additional groups not relevant for this review [14]

Included studies

- Cross-reference from other studies (*n*=1) [15]
- Identified from keeping up with literature (*n*=1) [16]

References

1. Farina A, Morano D, Arcelli D, De SP, Sekizawa A, Purwosunu Y, Zucchini C, Simonazzi G, Okai T, Rizzo N (2009) Gene expression in chorionic villous samples at 11 weeks of gestation in women who develop preeclampsia later in pregnancy: implications for screening. Prenat Diagn 29: 1038-1044.

2. Founds SA, Conley YP, Lyons-Weiler JF, Jeyabalan A, Hogge WA, Conrad KP (2009) Altered global gene expression in first trimester placentas of women destined to develop preeclampsia. Placenta 30: 15-24.

3. Herse F, Dechend R, Harsem NK, Wallukat G, Janke J, Qadri F, Hering L, Muller DN, Luft FC, Staff AC (2007) Dysregulation of the circulating and tissue-based renin-angiotensin system in preeclampsia. Hypertension 49: 604-611.

4. Eide IP, Isaksen CV, Salvesen KA, Langaas M, Schonberg SA, Austgulen R (2008) Decidual expression and maternal serum levels of heme oxygenase 1 are increased in pre-eclampsia. Acta Obstet Gynecol Scand 87: 272-279.

5. Lian IA, Toft JH, Olsen GD, Langaas M, Bjorge L, Eide IP, Bordahl PE, Austgulen R (2010) Matrix metalloproteinase 1 in pre-eclampsia and fetal growth restriction: reduced gene expression in decidual tissue and protein expression in extravillous trophoblasts. Placenta 31: 615-620.

6. Loset M, Mundal SB, Johnson MP, Fenstad MH, Freed KA, Lian IA, Eide IP, Bjorge L, Blangero J, Moses EK, Austgulen R (2011) A transcriptional profile of the decidua in preeclampsia. Am J Obstet Gynecol 204: 84.e1-27.

7. Toft JH, Lian IA, Tarca AL, Erez O, Espinoza J, Eide IP, Bjorge L, Draghici S, Romero R, Austgulen R (2008) Whole-genome microarray and targeted analysis of angiogenesis-regulating gene expression (ENG, FLT1, VEGF, PlGF) in placentas from pre-eclamptic and small-for-gestational-age pregnancies. J Matern Fetal Neonatal Med 21: 267-273.

8. Guller S, Buhimschi CS, Ma YY, Huang ST, Yang L, Kuczynski E, Zambrano E, Lockwood CJ, Buhimschi IA (2008) Placental expression of ceruloplasmin in pregnancies complicated by severe preeclampsia. Lab Invest 88: 1057-1067.

9. Hviid TV, Larsen LG, Hoegh AM, Bzorek M (2004) HLA-G expression in placenta in relation to HLA-G genotype and polymorphisms. Am J Reprod Immunol 52: 212-217.

10. Kivinen K, Peterson H, Hiltunen L, Laivuori H, Heino S, Tiala I, Knuutila S, Rasi V, Kere J (2007) Evaluation of STOX1 as a preeclampsia candidate gene in a population-wide sample. Eur J Hum Genet 15: 494-497.

11. Peterson H, Laivuori H, Kerkela E, Jiao H, Hiltunen L, Heino S, Tiala I, Knuutila S, Rasi V, Kere J, Kivinen K (2009) ROCK2 allelic variants are not associated with pre-eclampsia susceptibility in the Finnish population. Mol Hum Reprod 15: 443-449.

12. Anteby EY, Ayesh S, Shochina M, Hamani Y, Schneider T, Al-Shareef W, Hochberg A, Ariel I (2005) Growth factor receptor-protein bound 2 (GRB2) upregulation in the placenta in preeclampsia implies a possible role for ras signalling. Eur J Obstet Gynecol Reprod Biol 118: 174-181.

13. Jarvenpaa J, Vuoristo JT, Ukkola O, Hirvikoski P, Savolainen ER, Raudaskoski T, Ryynanen M (2008) Cord compression may rapidly influence the expression of placental angiogenic genes in pre-eclampsia. Placenta 29: 436-438.

14. Centlow M, Carninci P, Nemeth K, Mezey E, Brownstein M, Hansson SR (2008) Placental expression profiling in preeclampsia: local overproduction of hemoglobin may drive pathological changes. Fertil Steril 90: 1834-1843.

15. Pang ZJ, Xing FQ (2003) Comparative study on the expression of cytokine--receptor genes in normal and preeclamptic human placentas using DNA microarrays. J Perinat Med 31: 153-162.

16. Centlow M, Wingren C, Borrebaeck C, Brownstein MJ, Hansson SR (2011) Differential gene expression analysis of placentas with increased vascular resistance and pre-eclampsia using whole-genome microarrays. J Pregnancy 2011: 472354.
